# Variation in the Exon 3–4 Region of Ovine *KRT85* and Its Effect on Wool Traits

**DOI:** 10.3390/ani14152272

**Published:** 2024-08-05

**Authors:** Wenqiong Chai, Huitong Zhou, Hua Gong, Changfa Wang, Jon G. H. Hickford

**Affiliations:** 1 Liaocheng Research Institute of Donkey High-Efficiency Breeding and Ecological Feeding, Liaocheng University, Liaocheng 252059, China; chaiwenqiong@lcu.edu.cn (W.C.); wangcf1967@163.com (C.W.); 2 Gene-Marker Laboratory, Faculty of Agricultural and Life Science, Lincoln University, Lincoln 7647, Canterbury, New Zealand; huitong.zhou@lincoln.ac.nz (H.Z.); hua.gong@lincolnuni.ac.nz (H.G.)

**Keywords:** *KRT85*, variation, wool traits, sheep

## Abstract

**Simple Summary:**

α-keratins are the main structural proteins in the cortex of wool fibres. Variation in these proteins affects the structure and fibre characteristics of wool, making keratin genes good candidates for the development of gene markers to selectively breed for better wool. Variation was found in the keratin gene *KRT85*, with the presence of the *A* variant being associated with an increase in greasy fleece weight and clean fleece weight, and the presence of *B* associated with an increased wool prickle factor. This may assist in the development of gene markers to assist in sheep breeding.

**Abstract:**

α-keratins are structural proteins in the cortex of wool fibres and assemble in an organized fashion into keratin intermediate filaments. Variation in these keratin proteins affects the structure and characteristics of wool fibre, making keratin genes ideal candidates for the development of gene markers that describe variations in wool traits. A region of *KRT85* spanning exon 3–4 (including the entire exon 3, intron 3, exon 4 and part of intron 4) was investigated. Two banding patterns defining two variant sequences (*A* and *B*) were observed in this region, and these were characterised by the presence of two single nucleotide polymorphisms. The effect of this variation in the exon 3–4 region of *KRT85* on wool traits was investigated in 463 Merino × Southdown-cross lambs. The frequencies of these two variants in these sheep were 55.6% and 44.4%, respectively. Three different genotypes were observed with frequencies of 32.6%, 46.1% and 21.3% for *AA*, *AB* and *BB,* respectively. The presence of *A* was associated with an increase in greasy fleece weight and clean fleece weight, while the presence of *B* was associated with an increased wool prickle factor. These findings should be replicated in a broader range of sheep breeds to determine whether the associations are robust and to clarify whether the observed effects are attributable to breed differences or to gene effects themselves.

## 1. Introduction

The “hard” α-keratins contribute to the formation of keratinized structures in mammalian nails, hair and claws [1], and they are the major proteins in wool fibres. These keratins have been classified into two families, type I and type II keratins, with the type I keratins being acidic proteins and the type II keratins being basic-to-neutral proteins. To date, eleven functional type I and six type II keratin genes (*KRTs*) have been identified in human hair [1,2]. In sheep, 17 keratin genes have been identified, but their composition differs based on the absence of *KRT37* in the type I family and the presence of *KRT87* in the type II family [3].

Of the 54 keratin genes that have been characterized in humans, 17 are structurally classified as hair genes [3]. In sheep, the keratin genes are clustered at chromosomes 3q14-q22 and 11q25-q29 [4]. Of these, ten (*KRT31*, *KRT32*, *KRT33A*, *KRT33B*, *KRT34-36*, *KRT38-40*) encode type I keratins and seven (*KRT81-KRT87*) encode type II keratins [3,5,6,7].

The ovine keratin gene *KRT85* (ENSOARG00020013482) is expressed in both the fibre cuticle and cortex, along with *KRT32* and *KRT35* [3]. *KRT35* and *KRT85* are expressed earlier in fibre development than other *KRT*s. *KRT85* is located on chromosome 3, along with the other type II keratin genes, and positioned between nucleotides 134,015,188 and 134,022,879 of the forward strand of the ARS-UI_Ramb_v2.0:CM028706.1 genome construct. In human hair, K85 forms filaments with K36 in the hair matrix and keratin filaments with K31 in the lower portion of hair cortex [2].

Previous studies have suggested that the QTLs affecting wool traits are located on chromosome 3, with markers observed for greasy fleece weight (GFW) and the coefficient of the variation of fibre curvature (CVFD) [8,9,10]. However, only a small number of studies have described genetic variation in the chromosome 3 keratin genes. McLaren et al. (1997) reported two variants of *KRT83* using the polymerase chain reaction-restriction fragment length polymorphism (PCR-RFLP) typing method [11], and nine variants of *KRT87* (formerly *KRT2.13*) were reported by McKenzie et al. [12]. One, two and five variants have been described in the *KRT83* promoter, exon 2 and exon 3–4 regions using a PCR single-stranded conformational polymorphism (PCR-SSCP) approach, and the variation in the *KRT83* exon 3–4 region was reported to be associated with the mean fibre diameter (MFD) [13].

In this study, PCR-SSCP was used to investigate sequence variation in the exon 3–4 region of *KRT85*, and the associations, if any, that may occur between *KRT85* sequence variations and various commercially important wool traits.

## 2. Materials and Methods

### 2.1. Sheep Blood and Wool Samples

Approval for the present study was obtained from the Lincoln University Animal Ethics Committee under the provisions of the Animal Welfare Act 1999 (New Zealand Government). Initially, 300 test panel sheep from 26 farms, including Merino sheep (*n* = 100), New Zealand (NZ) Romney sheep (*n* = 100) and white Dorper sheep (*n* = 100), were screened to search for variation in the exon 3–4 region of *KRT85*. Subsequently, 463 Southdown × Merino-cross lambs from seven sire lines were used for the association studies. Blood samples for all the sheep were collected onto FTA cards (Whatman BioScience, Middlesex, UK), and then genomic DNA was extracted from the dried blood spots using a two-step procedure [14].

Wool samples were taken at 12 months of age from the mid-side of the sheep at shearing, and the entire fleece was weighed to give the GFW. Key commercial wool traits were then measured from the samples by the New Zealand Wool Testing Authority Ltd. (NZWTA, Napier, New Zealand) using the International Wool Testing Organization (IWTO)’s standardized methods (https://iwto.org/wool-supply-chain/wool-testing/; accessed 2 July 2024). This included the measurement of wool yield (Yield), MFD, fibre diameter standard deviation (FDSD), CVFD, mean fibre curvature (MFC), mean staple length (MSL), mean staple strength (MSS) and prickle factor (PF; the percentage of fibres of a diameter greater than 30 microns). The clean fleece weight (CFW) was calculated from the GFW and yield (CFW (kg) = GFW (kg) × Yield (%)).

### 2.2. PCR Amplification and Genotyping of the KRT85 Exon 3–4 Region

Based on the ovine *KRT85* sequence in the Texel sheep Ovine Genome Assembly v3 (GCF_000298735.1, GenBank), two PCR primers (5′-CTGACTCCTCAGCTCCAGC-3′ and 5′-CCCTCACCTCATCATAGAG-3′) were designed to amplify a 347 bp fragment spanning part of intron 2, exon 3, intron 3 and exon 4 of the gene. Amplifications were undertaken in 15 uL reactions that included the genomic DNA in a 1.2 mm spot of blood on the FTA cards, 10× reaction buffer, 0.5 U of Taq DNA polymerase (Qiagen, Hilden, Germany), 250 nM of each primer, 150 µM of each dNTP (Eppendorf, Hamburg, Germany) and a final Mg^2+^ concentration of 2.5 mM. The cycling parameters for PCR amplification consisted of an initial denaturation step at 94 °C for 2 min; followed by 35 cycles of 30 s at 95 °C, 30 s at 58 °C and 30 s at 72 °C; and a final extension at 72 °C for 5 min. Amplification was undertaken in S1000 thermal cyclers (Bio-Rad, Hercules, CA, USA).

To genotype the amplicons produced by the PCR amplifications, a PCR-SSCP technique was used. In these analyses, a 0.7 µL aliquot of PCR product was mixed with 7 µL of loading dye (98% formamide, 10 mM EDTA, 0.025% bromophenol blue, 0.025% xylene-cyanol), denatured at 95 °C for 5 min, and then cooled rapidly on wet ice. Electrophoreses were carried out in 0.5× TBE buffer in 14% polyacrylamide gels (37.5:1; Bio-Rad) for 18 h at 29 °C and 200 volts. Once reference samples were determined for each variant, they were included on all subsequent gels to facilitate the precise genotyping of all the sheep. All gels were silver-stained according to the method of Byun et al. [15]. Amplicons of the exon 3–4 variants were subsequently sequenced using the rapid approach described by Gong et al. [16]. 

### 2.3. Statistical Analyses

Statistical analyses were carried out and Minitab version 16. General Linear Mixed-effects Models (GLMMs) were used to assess the effect of the presence/absence of *KRT85* exon 3–4 variants on wool traits. In all the models, variant presence/absence, gender and birth rank were fitted as fixed factors, and sire line was fitted as a random factor.

Initially, ‘single-variant’ presence/absence models were created. Subsequently, any variant–wool trait association in the single-variant models with a *p*-value of <0.200 (and which could therefore potentially impact the wool trait being tested) were factored into ‘multi-variant models’, an approach that was used by Forrest et al. [17].

The model used was Y_jklm_ = µ + V1_j_ + V2_k_ + G_l_ + S_m_ + e_jklm_, where Y_jklm_ is the phenotypic value for the jklm^th^ sheep, µ is the group raw mean for the trait, V_j_ is the effect of the j^th^ variant (presence and absence), V2_k_ is effect of the second variant’s presence/absence (if factored in), G_l_ is the effect of gender, S_m_ is the effect of the m^th^ sire and e_jklm_ is the random residual effect. Significance was accepted at *p* < 0.05.

## 3. Results

### 3.1. Detection of Variation in Ovine KRT85 in the Exon 3–4 Region

Two PCR-SSCP gel banding patterns were observed with the conditions that were developed for the analysis of the *KRT85* exon 3–4 region, and the genotypes of the 300 test panel sheep and 463 Merino × Southdown-cross lambs could be determined. Upon sequencing, two variant sequences of this region of the gene were confirmed in the amplified region. These sequences were named variants *A* and *B*, and the sequences of the two variants have been deposited in GenBank and assigned the accession numbers MH910660 and MH910661.

The variant frequencies were 55.6% and 44.4% for *A* and *B*, respectively, in the Merino × Southdown-cross lambs. Despite the breed differences in the 300 test panel sheep, only these two variants were detected in that population too.

In the Merino × Southdown-cross lambs, three different genotypes were observed: *AA*, *AB* and *BB*, with frequencies of 32.6%, 46.1% and 21.3%, respectively. However, frequencies are not reported for the test panel sheep as they were selected to ascertain how much genetic variation may exist in this gene region, and not because they were representative of the different breeds.

**Figure 1:**
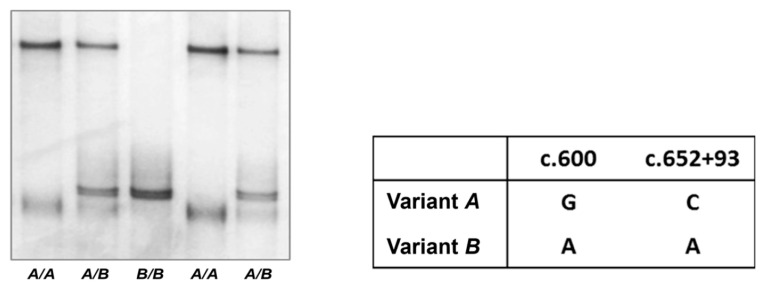
Variation identified in the ovine *KRT85* exon 3–4 region, with sequences deposited under GenBank Accession Nos. MH910660 (variant *A*) and MH910661 (variant *B*).

The sequence difference between *A* and *B* was because of the presence of two single nucleotide polymorphisms (SNPs; c.600G/A and c.652+93C/A) in the amplified region of *KRT85* (Figure 1), with one SNP located in exon 3 and the other in intron 3. The SNP located in exon 3 was synonymous and would not result in an amino acid change.

### 3.2. Associations between KRT85 Exon 3–4 Variants and Variation in Selected Wool Traits

The presence of *A* tended to be associated with increased GFW and CFW in the single-variant GLMMs, an association that became significant in the multi-variant GLMM (Table 1). The presence of *B* was associated with an increased PF (Table 1).

## 4. Discussion

Here, we report the identification of variation in the ovine *KRT85* exon 3–4 region. Of the two SNPs detected, one occurred in exon 3 and was synonymous, and the other was in a non-coding intron 3 region. Sulayman et al. [18] reported three nucleotide substitutions in a *KRT85* exon 3 fragment of Chinese Merino (Xinjiang type) sheep, with one being silent, one creating a nonsense mutation and one creating a synonymous mutation in the protein [18]. The variation described in the Chinese Merino seems to be greater than in all the New Zealand sheep tested, despite the diverse origins of these breeds, with the New Zealand Romney having its origin in British Romney Marsh sheep, the Merino coming from Spain, the Southdown being British and the Dorper having South African origins.

While none of the SNPs described here would appear to have major effects on the K85 protein, synonymous nucleotide variations are increasingly being recognized as a factor that can affect protein expression, such as by altering transcription, mRNA half-life, translation, protein stability and protein function. To illustrate this point, a synonymous variant of the human corneodesmin gene (*CSDN*) caused an increase in mRNA half-life and was associated with a psoriasis phenotype [19], but its mechanism of action could not be ascertained. In both that study and herein, the silent SNPs may be linked to a nucleotide variation upstream or downstream in an extended haplotype that affects the regulation of gene expression. Equally, while the intron variation might be benign, it is now widely understood that introns impact gene expression, mRNA splicing, mRNA stability and can optimise transcriptional efficiency in their host gene. That is, the presence of intronic mutations can affect regulatory expression elements from transcription to translation [20].

The genetic structure of complex traits, especially the number of loci affecting the trait and the distribution of their effects, is still largely unresolved. Accordingly, further studies are required to assess the effect of polymorphisms in both coding and non-coding regions of candidate genes and their association with mRNA levels, translation, protein processing, the activity of biochemical pathways and the tissue(s) in which the gene is functional. Hence, in this study, it might be more prudent to conclude that *KRT85* variation would have a functional impact, until proven otherwise.

Sulayman et al. reported an association between a *KRT85* exon 3 fragment and wool traits in Chinese Merino sheep, with the JJ genotype sheep having a significantly greater wool crimp score, body size and fibre diameter than the other genotypes [18]. The associations detected in the present study are hard to compare with these findings, with variant *B* of the *KRT85* exon 3–4 region only being associated with PF in the single-variant models, and with the two fleece weight traits CFW and GFW in the multi-variant models. Prickle factor is related to MFD (higher-diameter fibres impart more prickle), but no effect was observed with *KRT85* variation and MFC, despite crimp and MFC being phenotypically correlated [21]. 

The observation that both CFW and GFW were associated with variations in *KRT85* is consistent with the strong positive correlation reported between these traits [22]. It is also notable that variant *A*, which is associated with CFW and GFW, was also trending towards an association with MSL. Intuitively, one might expect longer fibres to suggest greater wool growth and hence increased CFW and GFW, and, in that respect, Gong et al. have reported that GFW and CFW are both moderately positively correlated with an increased MSL [22].

*KRT85* is located on the same chromosome as *KRT83*, but variation in the exon 3–4 fragment of *KRT83* was associated with MFD, whereas variation in the exon 3–4 fragment of *KRT85* was found to be associated with increased GFW and CFW. This suggests that the two genes have independent effects. Gong et al. [22] reported no significant correlation between GFW and CFW and MFD, with this supporting the argument that *KRT83* and *KRT85* function independently.

The associations detected in this study are consistent with the findings that revealed a QTL for GFW on chromosome 3 [23]. The effect of variation in the *KRT85* exon 3–4 fragment on wool traits is also similar to that of *KRTAP1-2* [22], *KRTAP6-1* [24] and *KRTAP22-1* [25], despite these *KRTAPs* being located on different chromosomes. Once again, this suggests that wool traits are not the consequence of the action of individual *KRTs* or *KRTAPs* [26], but instead reflect the coordinated expression of many genes.

## 5. Conclusions

The present research identified variation in the exon 3–4 region of the keratin gene *KRT85*, with the presence of the *A* variant being associated with an increase in GFW and CFW, and the presence of *B* associated with an increased wool PF in 463 Merino × Southdown-cross lambs. This work needs to be replicated in other sheep breeds to ascertain whether this association is observed elsewhere, and this may then assist in the development of gene markers for sheep breeding.

## Figures and Tables

**Table 1:** Association of *KRT85* exon 3–4 variants with selected wool traits.

Trait ^2^	Variant			Single-Variant Model			Multi-Variant Model			
		Absent (n)	Present (n)	Absent (Mean ± SEM ^1^)	Present (Mean ± SEM)	*p*	Other Variant Fitted	Absent (Mean ± SEM)	Present (Mean ± SEM)	*p*
GFW	*A*	117	346	2.2 ± 0.10	2.3 ± 0.09	0.282	*B*	**2.4 ± 0.09**	**2.5 ± 0.09**	**0.045**
(kg)	*B*	105	358	*2.2 ± 0.10*	*2.3 ± 0.09*	*0.110*				
CFW	*A*	117	346	*1.7 ± 0.08*	*1.7 ± 0.08*	*0.105*	*B*	**1.7 ± 0.07**	**1.8 ± 0.06**	**0.024**
(kg)	*B*	105	358	*1.7 ± 0.08*	*1.7 ± 0.08*	*0.142*	*A*	1.7 ± 0.07	1.8 ± 0.06	0.305
YIELD	*A*	117	346	75.3 ± 1.46	76.1 ± 1.38	0.301				
(%)	*B*	105	358	76.0 ± 1.50	75.8 ± 1.37	0.869				
MFD	*A*	117	346	18.9 ± 0.44	18.9 ± 0.41	0.830				
(µm)	*B*	105	358	18.7 ± 0.45	18.9 ± 0.41	0.349				
FDSD	*A*	117	346	4.1 ± 0.16	4.1 ± 0.15	0.417	*B*	4.1 ± 0.11	4.1 ± 0.10	0.562
(µm)	*B*	105	358	*4.0 ± 0.16*	*4.1 ± 0.15*	*0.100*				
CVFD	*A*	117	346	*21.8 ± 0.61*	*21.3 ± 0.58*	*0.151*	*B*	21.8 ± 0.42	21.5 ± 0.37	0.265
	*B*	105	358	*21.1 ± 0.63*	*21.5 ± 0.57*	*0.178*	*A*	21.5 ± 0.42	21.8 ± 0.37	0.233
MSL	*A*	117	346	*81.1 ± 2.90*	*83.3 ± 2.75*	*0.130*				
(mm)	*B*	105	358	81.7 ± 3.00	82.7 ± 2.73	0.467	*A*	83.0 ± 1.97	84.0 ± 1.74	0.416
MSS	*A*	117	346	25.3 ± 2.09	24.3 ± 1.99	0.341				
(N/ktex)	*B*	105	358	24.9 ± 2.16	24.6 ± 1.97	0.759				
PF	*A*	117	346	2.0 ± 0.77	2.0 ± 0.73	0.943	*B*	1.8 ± 0.67	1.9 ± 0.61	0.787
(%)	*B*	105	358	**1.3 ± 0.79**	**2.1 ± 0.72**	**0.032**				
MFC	*A*	117	346	89.4 ± 3.55	90.9 ± 3.37	0.415				
(o/mm)	*B*	105	358	*90.5 ± 3.66*	*90.4 ± 3.34*	*0.964*				

^1^ Estimated marginal means and standard errors of the mean (SEM) derived from the GLMMs. *p* < 0.05 are in bold, and 0.05 ≤ *p* < 0.20 are italicized. ^2^ GFW—greasy fleece weight; CFW—clean fleece weight; MFD—mean fibre diameter; CVFD—coefficient of variation of fibre diameter; FDSD—fibre diameter standard deviation; MSS—mean staple strength; MSL—mean staple length; PF—prickle factor (percentage of fibres over 30 μm); MFC—mean fibre curvature.

## Data Availability

The raw data supporting the conclusions of this article are available on request.

## References

[1] Langbein L., Rogers M.A., Winter H., Praetzel S., Beckhaus U., Rackwitz H.R., Schweizer J. (1999). The catalog of human hair keratins. I. Expression of the nine type I members in the hair follicle. J. Biol. Chem..

[2] Langbein L., Rogers M.A., Winter H., Praetzel S., Schweizer J. (2001). The catalog of human hair keratins. II. Expression of the six type II members in the hair follicle and the combined catalog of human type I and II keratins. J. Biol. Chem..

[3] Yu Z., Wildermoth J.E., Wallace O.A.M., Gordon S.W., Maqbool N.J., Maclean P.H., Nixon A.J., Pearson A.J. (2011). Annotation of sheep keratin intermediate filament genes and their patterns of expression. Exp. Dermatol..

[4] Hediger R., Ansari H.A., Stranzinger G.F. (1991). Chromosome banding and gene localizations support extensive conservation of chromosome structure between cattle and sheep. Cytogenet. Cell Genet..

[5] Powell B., Crocker L., Rogers G. (1992). Hair follicle differentiation: Expression, structure and evolutionary conservation of the hair type II keratin intermediate filament gene family. Development.

[6] Powell B.C., Crocker L.A., Rogers G.E. (1993). Complete sequence of a hair-like intermediate filament type II keratin gene. Mitochondrial DNA.

[7] Wilson B.W., Edwards K., Sleigh M., Byrne C., Ward K. (1988). Complete sequence of a type-I microfibrillar wool keratin gene. Gene.

[8] Allain D., Lantier I., Elsen J.M., François D., Brunel J.C., Weisbecker J.L., Schibler L., Vaiman D., Cribiu E., Gautier A. A design aiming at detecting QTL controlling wool traits and other traits in the INRA401 sheep line. Proceedings of the World Congress Genetics Applied To Livestock Production.

[9] Allain D., Miari S., Usai M.G., Barillet F., Sechi T., Sechi S., Carta A. SNP mapping of QTL affecting wool traits in a sheep backcross Sarda x Lacaune resource population. Proceedings of the 64th annual meeting of the European Federation of Animal Science.

[10] Ponz R., Moreno C., Allain D., Elsen J.M., Lantier F., Lantier I., Brunel J.C., Pérez-Enciso M. (2001). Assessment of genetic variation explained by markers for wool traits in sheep via a segment mapping approach. Mamm. Genome.

[11] McLaren R.J., Rogers G.R., Davies K.P., Maddox J.F., Montgomery G.W. (1997). Linkage mapping of wool keratin and keratin-associated protein genes in sheep. Mamm. Genome.

[12] McKenzie G.W., Arora R., Hickford J.G. (2012). Genetic variation in the 5′UTR of the KRT2.13 gene of sheep. Anim. Sci. J..

[13] Chai W., Zhou H., Forrest R.H., Gong H., Hodge S., Hickford J.G. (2017). Polymorphism of KRT83 and its association with selected wool traits in Merino-cross lambs. Small Rumin. Res..

[14] Zhou H., Hickford J.G., Fang Q. (2006). A two-step procedure for extracting genomic DNA from dried blood spots on filter paper for polymerase chain reaction amplification. Anal. Biochem..

[15] Byun S.O., Fang Q., Zhou H., Hickford J.G.H. (2009). An effective method for silver-staining DNA in large numbers of polyacrylamide gels. Anal. Biochem..

[16] Gong H., Zhou H., Plowman J.E., Dyer J.M., Hickford J.G. (2010). Analysis of variation in the ovine ultra-high sulphur keratin-associated protein KAP5-4 gene using PCR-SSCP technique. Electrophoresis.

[17] Forrest R.H., Itenge-Mweza T.O., McKenzie G.W., Zhou H., Frampton C.M., Hickford J.G. (2009). Polymorphism of the ovine β 3-adrenergic receptor gene (ADRB3) and its association with wool mean staple strength and yield. Anim. Genet..

[18] Sulayman A., Tursun M., Sulaiman Y., Huang X., Tian K., Tian Y., Xu X., Fu X., Mamat A., Tulafu H. (2018). Association analysis of polymorphisms in six keratin genes with wool traits in sheep. Asian-Australas. J. Anim. Sci..

[19] Capon F., Allen M.H., Ameen M., Burden A.D., Tillman D., Barker J.N., Trembath R.C. (2004). A synonymous SNP of the corneodesmosin gene leads to increased mRNA stability and demonstrates association with psoriasis across diverse ethnic groups. Hum. Mol. Genet..

[20] Hir H.L., Nott A., Moore M.J. (2003). How introns influence and enhance eukaryotic gene expression. Trends Biochem. Sci..

[21] Yu H., Hurren C., Liu X., Wang X. (2022). Understanding the difference in softness of Australian Soft Rolling Skin wool and conventional Merino wool. Text. Res. J..

[22] Gong H., Zhou H., Hodge S., Dyer J.M., Hickford J.G. (2015). Association of wool traits with variation in the ovine KAP1-2 gene in Merino cross lambs. Small Rumin. Res..

[23] Allain D., Schibler L., Mura L., Barillet F., Sechi T., Rupp R., Casu S., Cribiu E., Carta A. QTL detection with DNA markers for wool traits in a sheep backcross Sarda × Lacaune resource population. Proceedings of the World Congress on Genetics Applied To Livestock Production.

[24] Zhou H., Gong H., Li S., Luo Y., Hickford J.G.H. (2015). A 57-bp deletion in the ovine KAP6-1 gene affects wool fibre diameter. J. Anim. Breed. Genet..

[25] Li S., Zhou H., Gong H., Zhao F., Wang J., Liu X., Luo Y., Hickford J.G. (2017). Identification of the Ovine Keratin-Associated Protein 22-1 (KAP22-1) Gene and Its Effect on Wool Traits. Genes.

[26] Zhou H., Gong H., Wang J., Luo Y., Li S., Tao J., Hickford J.G.H. (2021). The complexity of the ovine and caprine keratin-associated protein genes. Int. J. Mol. Sci..

